# Multi-Scale Structures and Functional Properties of Quinoa Starch Extracted by Alkali, Wet-Milling, and Enzymatic Methods

**DOI:** 10.3390/foods11172625

**Published:** 2022-08-30

**Authors:** Shahid Ahmed Junejo, Jun Wang, Ying Liu, Rui Jia, Yibin Zhou, Songnan Li

**Affiliations:** 1 State Key Laboratory of Food Nutrition and Safety, Tianjin University of Science and Technology, Tianjin 300457, China; 2 State Key Laboratory of Tea Plant Biology and Utilization, Anhui Agricultural University, 130 Changjiang West Road, Hefei 230036, China; 3 Joint International Research Laboratory of Agriculture and Agri-Product Safety, The Ministry of Education of China, Institutes of Agricultural Science and Technology Development, Yangzhou University, Yangzhou 225009, China; 4 School of Food Science and Engineering, South China University of Technology, Guangzhou 510640, China; 5 School of Tourism and Cuisine, Yangzhou University, Yangzhou 225127, China; 6 Key Laboratory of Agricultural Products Processing Engineering of Anhui Province, School of Tea and Food Technology, Anhui Agricultural University, Hefei 230036, China

**Keywords:** quinoa starch, extraction methods, multi-scale structures, functional properties, *in vitro* digestion

## Abstract

The purpose of this study is to investigate the effects of starch extraction methods (alkali, wet-milling, and enzymatic) on the multi-scale structures and functional properties of quinoa starch. When the enzymatic method was compared with alkali and wet-milling, it showed higher protein content (2.4%), larger mean size of aggregated granules (44.1 μm), higher relative crystallinity (29.0%), scattering intensity (17.8 α.u.), absorbance ratio of 1047/1022 (0.9), single and double helical content (8.2% and 23.1%), FWHM ratio (1.5), and average molecular weight and radius of gyration (1.58 × 10^7^ g/mol and 106.8 nm), respectively. Similarly, quinoa starch by enzymatic extraction had a higher onset (82.1 °C), peak (83.8 °C), and conclusion (86.3 °C) temperatures, as well as an enthalpy change (6.8 J/g). It further showed maximum hardness (238.8 N), gumminess (105.6 N), chewiness (80.2 N), SDS content (7.5% of raw and 4.8% of cooked), and RS content (15.4% of raw and 13.9% of cooked), whereas it contained minimum RDS content (77.1% of raw and 81.9% of cooked). The results suggest that extraction of starch by the enzymatic method could be a viable approach to retain the native structure of starch and may eventually improve the glycemic response.

## 1. Introduction

Quinoa (*Chenopodium Quinoa Willd.*) seeds are pseudo-cereals of the Chenopodiaceae family with dicotyledon plants and are widely grown as an edible and staple grain crop in South America [1]. Quinoa seeds are a useful source of starch, protein, fats, dietary fiber, minerals, vitamins, and polyphenols. The major component of quinoa seed is starch (∼30–70%), with small granule sizes (~1–3 μm) and amylose content (∼0.3–27.7%) [2]. Quinoa starch amylose is less branched, but amylopectin has a high proportion of short chains and fingerprint A-chains [3]. These structural characteristics of quinoa starch have led to some unique properties, including low gelatinization temperature and gel strength, high enzyme susceptibility, Pickering emulsifying property, and targeted delivery function [4]. Quinoa starch provides more than half of the energy, and its slow-digesting properties are nutritionally desirable. Thus, it can be used as a novel source for expanding food functional properties (i.e., texture, sensory attributes, and nutritional value).

Starch is an important raw material used in food (thickener, stabilizer, and weaning foods) and non-food industries (pharmaceuticals and textiles). The world’s annual production of starch is estimated at about 60 million tons [5], which is extracted from cereals, tubers, and root crops. Its 60% is used in foods (e.g., bakery products, soups, sauces, ice cream, sugar syrups, snack foods, baby foods, meat products, drinks, and fat substitutes), while the remaining 40% is used in pharmaceuticals and non-edible applications [6]. The various extracting techniques (i.e., alkali, wet-milling, and enzymatic method) are used with different processing conditions and chemicals to obtain starch, which dominates the chemical composition and multi-scale structure of isolated starch [7,8], resulting in desirable and undesirable effects on their physicochemical and functional properties. Alkali is the process of starch extraction that involves the steeping, physical shearing, and slightly high pH value induced by sodium hydroxide, which affects the multi-scale structure and causes changes in physical and chemical properties, improves the solubility of starch and protein, degrades the starch molecules through attacking functional groups, and reduces molecular weight [9,10,11]. Meanwhile, in the entire process of wet-milling extraction, large and long chains of molecules are either extracted poorly or are degraded easily. The sodium pyrosulfite in the soaking increases the water diffusion rate, disintegrates the matrix of protein-starch, and slows down the growth of microorganisms [12], whereas enzymatic extraction requires less grinding and smaller energy inputs, and also releases starch content from cell wall components with better recovery.

Since the methods of extraction induce structural differences among isolated starches, such changes may eventually affect the functional properties and lead to different industrial applications [7]. Therefore, three kinds of starch extraction techniques (i.e., alkali, wet-milling, and enzymatic method) were used to isolate quinoa starch, their basic composition (total starch, moisture, protein, lipid, and ash content), multi-scale structural characterization (scanning electron microscopy, particle size analyzer, X-ray diffraction, small-angle X-ray scattering analysis, attenuated total reflectance-fourier transformed infrared spectroscopy, laser confocal micro-Raman analysis, solid-state ^13^C nuclear magnetic resonance spectroscopy, and size exclusion chromatography) and functional properties (gelatinization, texture, and *in vitro* digestion) were explored, and their structure–function relationship was further studied. This study paves the way for designing a targeted functional starch source via the extraction technique of selection and optimization.

## 2. Materials and Methods

### 2.1. Materials

Quinoa seeds of good quality were provided by the Qinghai Farmer Co., Ltd. (Qinghai province, China). The protease of *streptomyces griseus* (type XIV), porcine pancreas α-amylase (P7545, 8 × USP), amyloglucosidase (A7095, ≥300 units/mL) enzymes, and glucose oxidase–peroxidase (GOPOD) were provided by Sigma-Aldrich Ltd. (St. Louis, MO, USA). The total starch testing assay kit (K-TSTA) was obtained from Megazyme International Ltd. (Bray, Co., Wicklow, Ireland). Other utilized chemicals or reagents are analytical grade.

### 2.2. Extraction of Starch Using the Alkali, Wet-Milling, and Enzymatic Methods

Quinoa starch was isolated using the alkali method as reported by Li, et al. [13] with slight modification. Quinoa seeds were ground into flour and dispersed in sodium hydroxide (0.3%) in a ratio of 1:5 (*w*/*w*), mixed, and left for 2 h. The mixture was passed through a mesh sieve with an opening of 0.18 mm. The milky starch precipitated and the upper supernatant layer was gradually poured out. The precipitated starch residues were thoroughly washed with distilled water and freeze-dried for further use. 

The wet-milling method was used to isolate quinoa starch following the procedure described previously [14] with modification. Quinoa seeds (50 g) were soaked in a 0.45% sodium pyrosulfite solution (150 mL, *w*/*v*) overnight at 4 °C and ground by a kitchen blender (SMF01, SUPOR, Zhejiang Supor Co., Ltd., Hangzhou, China). The sodium pyrosulfite solution (350 mL) was added to the ground mixture and stirred continuously to separate proteins and lipids, then centrifuged at 3000× *g* for 10 min. This process was repeated at least 3 times. The resulting fractions were further purified (10 times) by sodium chloride-toluene (1: 9, *w*/*v*) solution. Starch residues were further thoroughly washed with ethanol and distilled water until the toluene odor was removed completely. Then it was filtered, rinsed again with ethanol and distilled water through cheesecloth, and freeze-dried for 48 h.

Isolation of quinoa starch was performed by an enzymatic method (protease) using the procedure described by Zhao, et al. [15]. The quinoa seeds were ground by a kitchen grinder (SMF01, SUPOR, Zhejiang Supor Co., Ltd., Hangzhou, China), thoroughly mixed by hand, and then passed through a mesh sieve opening of 75 μm. The sieved sample (1.5 g) was soaked in 40 mL of sodium bisulfite solution (0.45%, *w*/*v*) for 18 h at room temperature and centrifuged at 4000× *g* for 10 min. The upper yellowish layer (protein and fiber) of the supernatant was discarded, and the residues were deproteinized by a protease enzyme (2.5 units/mL) in tricine buffer at 37 °C overnight. The residues were further thoroughly washed several times with absolute ethanol (40 mL) and distilled water (40 mL) to remove soluble impurities (e.g., amino acids and lipids). The pure odorless starch residues were freeze-dried (BTP 9ES, SP Scientific Industries, Inc., Warminster, PA, USA) for 48 h.

### 2.3. Chemical Composition Analysis

The extracted starch samples were determined for chemical composition analysis. Total starch was quantified using a starch assay kit according to the manufacturer’s instructions. Total protein was obtained using the Kjeldahl instrument (VELP Scientifica UDK159, Milan, Italy), with protein-to-nitrogen conversing factor (N × 6.25). The moisture, ash, and lipid contents were measured using American Association of Cereal Chemists (AACC) reported protocols [16].

### 2.4. Multi-Scale Structure Analyses

#### 2.4.1. Particle Size Analysis

Mastersizer 2000 (Malvern Instruments Co. Ltd., Solihull, UK) was used for particle size analysis according to a previous method [14]. Sample (1 g) was dispersed in distilled water (20 mL). The suspension was added drop-by-drop into the dispersion unit until it achieved 12% obscuration. The software recorded the mean diameter (D_50_), volume-weighted mean diameter (D_[4, 3]_), and surface-weighted mean diameter (D_[3, 2]_) of particle size in the range of 0.02 to 2000 μm, respectively.

#### 2.4.2. Scanning Electron Microscopy (SEM)

Quinoa starch samples isolated by different extraction methods were pasted on an adhesive carbon tape followed by platinum coating with a sputter coater (Cressington Scientific Instruments, Cressington, UK). Sample scanning was performed at an accelerated voltage of 10 kV using the SEM (Carl ZEISS, EVO 18, Oberkochen, Germany) and images were snapped at 500×, 1000×, and 10,000× magnifications.

#### 2.4.3. X-ray Diffraction (XRD)

The XRD patterns of the extracted starches were achieved by using D8 Advance (Bruker, Berlin, Germany). The diffractometer was set to 40 kV, 30 mA, and γ = 0.154 nm Cu Kα radiation. Scanning was performed at 0.5°/min from 4°–35° 2*θ*. The Jade software 6.5 version (Materials Data Inc., Livermore, CA, USA) was accessed to calculate the relative crystallinity as the ratio of peaks to the diffractogram area.

#### 2.4.4. Small-Angle X-ray Scattering (SAXS) Analysis

The SAXS system (Anton Paar, Graz, Austria) analysis was performed at 40 kV, 50 mA, and Cu-Ka radiation with an X-ray wavelength of 0.1542 nm. The equilibrated (at 20 °C for 24 h) starch samples (60% moisture) were exposed to the X-ray pasting cell for 10 min [17]. The scattering data was recorded using the IP Reader software program, and the obtained data was further processed by the SAXS quant software 3.0 version (Bruker, Berlin, Germany).

#### 2.4.5. Attenuated Total Reflectance-Fourier Transformed Infrared (ATR-FTIR) Spectroscopy

A spectrometer (Nicolet IS50, Thermo Fisher, Waltham, MA, USA) was used to determine the ATR-FTIR spectra according to the method reported by Mutungi et al. [18]. Sample scanning was performed automatically in the range from 1200 to 800 cm^−1^ spectrum baseline at 4 cm^−1^ resolution with an accumulation of 64 scans. The absorbance ratios (1047, 1022, and 998 cm^−1^) were recorded to analyze the short-range structures of starch.

#### 2.4.6. Solid-State ^13^C Nuclear Magnetic Resonance (NMR) Spectroscopy

Samples were analyzed for NMR by a spectrometer (Bruker Advance III HD 400, Bruker, Berlin, Germany) at a frequency of 400 MHz. The sample (200 mg, dry starch basis) was loaded into a cylindrical partially stabilized zirconium oxide (PSZ) rotor with a diameter of 4 mm and spun at 5 kHz. The acquisition time was 50 ms with the accumulation of 2400 scans per spectrum. The NMR spectroscopy results were analyzed in accordance with the previous report [19]. The sample was boiled for 1 h and frozen immediately using liquid nitrogen to prevent retrogradation. The boiled sample was tested as an amorphous reference after XRD detection. The Peakfit software 4.0 version (Systat Software, San Jose, CA, USA) was acquired to analyze spectral peaks.

#### 2.4.7. Laser Confocal Micro-Raman (LCM-Raman) Analysis

The Raman spectrometer equipped with a Leica microscope (Renishaw, Gloucestershire, UK) was used to analyze Raman spectra in extracted starches. The Raman system was calibrated at 532 cm^−1^ by using a silicon semiconductor [20]. Sample spectra were scanned in the range of 3500–0 cm^−1^ at a resolution of 5 cm^−1^. The starch molecular order was characterized by the full-width at half-maximum (FWHM) band at 480 cm^−1^ using the WIRE software 2.0 version (Wire Swiss GmbH, Zug, Switzerland).

#### 2.4.8. Gel Permeation Chromatography Coupled with Multi-Angle Light Scattering (GPC-MALS)

The analysis was conducted using the GPC system (Waters, Milford, MA, USA) equipped with a MALS detector (Wyatt Technology Co., Santa Barbara, CA, USA), a refractive index detector and 2 GPC columns (Styragel HMW 7 DMF 7.8 mm × 300 mm, Styragel HMW 6E DMF 8 mm × 300 mm) detector to measure the weight-average molecular weight (Mw) and the molar mass distribution of starch samples according to the reported method [21]. The starch sample (6 mg) was accurately weighed, incubated in 1.5 mL DMSO (mobile phase filtered at 0.45 µm using a hydrophilic Teflon filter) at 80 °C for 24 h, and centrifuged at 4000× *g* for 10 min. The absolute ethanol (10 mL) was further added twice to the precipitate sample, again dissolved in DMSO (2 mg mL^−1^), and transferred to GPC phials. The sample volume (100 µL) was injected with a flow rate of 1.0 mL/min at 50 °C. Astra V software (Astra, lnc., Quezon, Philippines) was used to calculate M_w_ with Zimm model.

### 2.5. Functional Properties

#### 2.5.1. Differential Scanning Calorimetry (DSC)

The DSC (Mettler Toledo, Schwerzenbach, Switzerland) was used to determine the thermal properties of extracted starches. The dried starch sample (3 mg) and distilled water (7 µL) were weighed accurately in a DSC pan, sealed hermetically, equilibrated at 25 °C overnight, and scanned at a 10 °C/min heating rate from 30–120 °C. The temperatures (onset, *T*_o_; peak, *T*_p_; and conclusion, *T*_c_) and enthalpy change (Δ*H*) were calculated by using STARe software (Version 15.0, Mettler Toledo, Schwerzenbach, Switzerland).

#### 2.5.2. Textural Analysis

The TA.XT Plus Texture Analyzer (Stable Micro Systems, Godalming, UK) equipped with a 36 mm diameter cylinder probe was operated at 50% compression rate and 1 mm/s speed to analyze textural analysis. The hardness, springiness, gumminess, chewiness, and resilience were analyzed by exponent software version 6 (Stable Micro Systems, Godalming, UK) according to the reported method [22].

#### 2.5.3. *In Vitro* Digestion of Starch

Quinoa starch isolated by different extraction methods was subjected to cooking (100 mg dried starch in 5 mL distilled water at 100 °C for 30 min) followed by freeze-drying for 48 h. Raw (uncooked) and cooked starch samples were analyzed for *in vitro* digestion according to the previous method [23]. A dry starch sample (100 mg) was incubated at 37 °C in a sodium acetate buffer solution (10 mL, 0.2 M, pH 5.2) containing porcine pancreatic α-amylase (290 units/mL and amyloglucosidase (15 units/mL). The digesting aliquots (0.5 mL) were taken at different time intervals (0, 20, and 120 min) and combined with absolute ethanol (2 mL) to deactivate the enzyme. The amount of glucose released from the extracted starch was quantified by the GOPOD assay kit. The percentage of starch hydrolyzed was calculated as the multiplication of obtained glucose with a 0.9 factor. The contents of the rapidly digestible starch (RDS), slowly digestible starch (SDS), and resistant starch (RS) were obtained by the values of G20 (glucose released within 20 min) and G120 (glucose released within 120 min) as reported previously [24].

### 2.6. Statistical Analysis

Analyses were performed by the statistical product and service solution (SPSS, version 21.0, Inc., Chicago, IL, USA). The one-way analysis of variance (ANOVA) was conducted using Duncan’s test with a significant difference level (*p* ≤ 0.05). All results were shown of double experiments with their means and standard deviations, and graphs were plotted by Origin 2019b.

## 3. Results and Discussion

### 3.1. Proximal Composition

The proximate composition of quinoa starch extracted by three different methods (alkali, wet-milling, and enzymatic) is shown in Table 1. Generally, the lowest amount of lipid, protein, and mineral content obtained by an extraction method is considered the most efficient method of starch extraction, purification, and with a better product [25]. The alkali, wet-milling, and enzymatic methods exhibited no significant effect on the total starch content (98.7%, 98.6%, and 97.1%), whereas the protein (2.4%), lipid (0.5%), and ash (0.2%) contents of the enzymatic method were observed to be higher than alkali and wet-milling, and the alkali method showed the lowest amount of protein (0.6%), lipid (0.5%), and ash (0.1%) contents. It indicated that during the alkali extraction method, starch granules were steeped in sodium hydroxide (0.3% *w/v*), which effectively solubilized the protein matrix and largely removed the protein, the results are consistent with a previous report [25]. Moreover, a similar study conducted on tef starch extraction reported that milled tef grain steeped in sodium hydroxide produced the highest starch purity (95.0%) with the lowest protein content (0.6%) [26].

### 3.2. Multi-Scale Structure Characteristics

#### 3.2.1. Starch Granule Morphology

**Figure 1:**
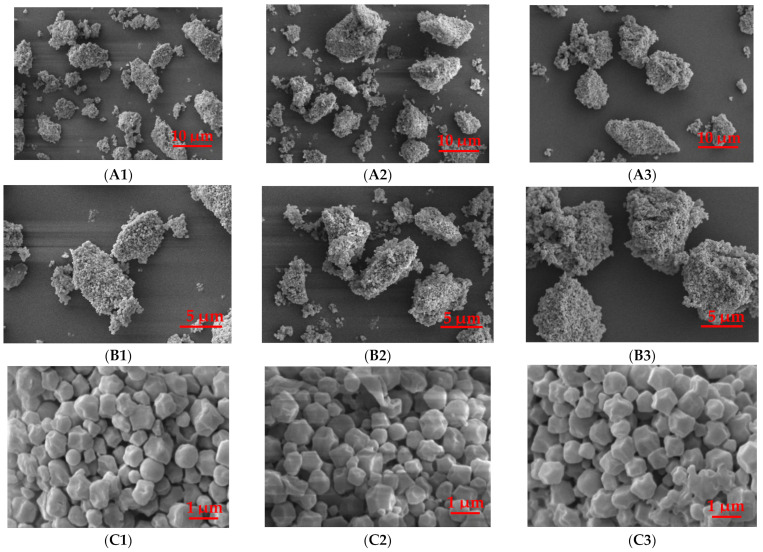
SEM images of quinoa starch isolated by alkali (**A1**–**C1**), wet-milling (**A2**–**C2**), and enzymatic (**A3**–**C3**) methods at 500×, 1000×, and 10000× magnifications.

The SEM micrographs and size distribution of quinoa starch extracted by three different methods (alkali, wet-milling, and enzymatic) are shown in Figure 1 and Table 1. The quinoa starch extracted by alkali, wet-milling, and enzymatic methods showed polygonal, angular, and irregular shapes of granules with no differences in their morphological structure, whereas the D_50_ (27.6, 34.9, and 44.1 μm), D_[4, 3]_ (34.5, 39.9 and 47.7 μm), and D_[3, 2]_ (6.2, 7.4 and 11.3 μm), respectively, showed significant differences. The size distribution of starch granules extracted by alkali, wet-milling, and enzymatic methods was observed higher (Figure 1 and Table 1) than previously suggested [2], possibly due to the aggregation of starch granules, evidenced by SEM images. A study conducted by Yu et al. on octenylsuccinylated taro starches suggested that the higher granule size could be due to the higher degree of aggregation of hydrophobically modified taro starch granules [27]. The enzymatic method showed the largest D_50_, D_[4, 3]_, and D_[3, 2]_ as compared to alkali and wet-milling. The higher D_50_, D_[4, 3]_, and D_[3, 2]_ of the enzymatic method suggests that the presence of sodium bisulfite in the enzymatic method facilitated the procurement of large starch granules [28], and could not effectively purify starch granules, while the lower D_50_, D_[4, 3]_, and D_[3, 2]_ of alkali and wet-milling methods indicated that sodium hydroxide present in the alkali method helped to penetrate water into starch followed by granule swelling and degraded amylose and amylopectin at the surface of the starch granules [29,30], whereas the wet-milling process also caused the loss of starch granules and ultimately changed the granule size distribution [9]. Moreover, Peng et al. compared the physicochemical properties and *in vitro* digestibility of different quinoa varieties and observed that quinoa starch was damaged due to different starch isolating methods [31].

#### 3.2.2. Crystalline Structure

**Figure 2:**
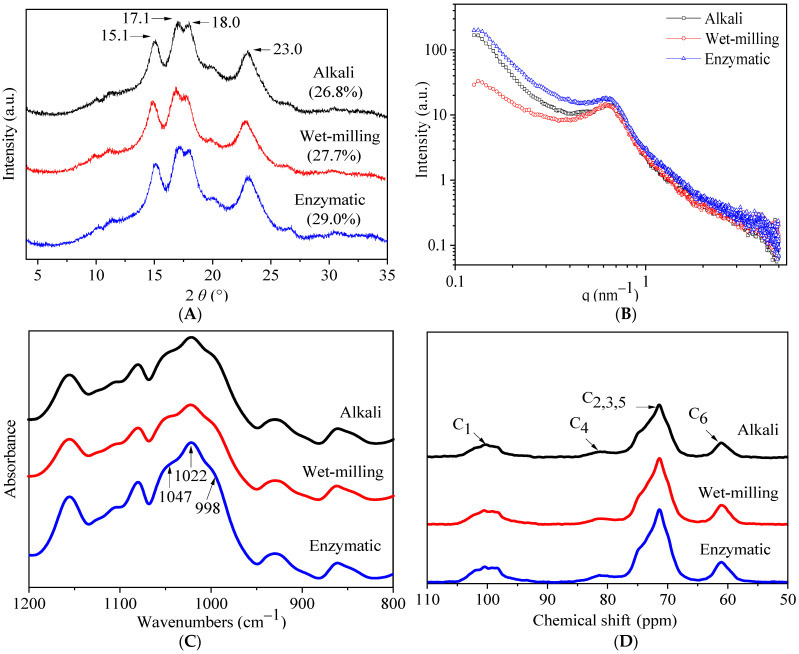
XRD (**A**), SAXS (**B**), FTIR (**C**), and NMR (**D**) spectrum of quinoa starch isolated by three different methods (alkali, wet-milling, and enzymatic). The XRD values in parentheses show the relative crystallinity of quinoa starch.

In Figure 2A and Table 2, the crystalline structure and relative crystallinity of quinoa starch extracted by alkali, enzymatic, and wet-milling methods are presented. The semi-crystalline system of starch granules is based on crystalline and amorphous regions. The XRD diffractogram exhibited the A-type structure patterns of quinoa starch samples with major peaks at 15.1°, 17.1°, 18.0° and 23.0° 2*θ*, consistent with previous reports [1,2]. All the extraction methods showed similar diffraction peaks with slightly lower peak intensity in alkali and wet-milling, whereas the relative crystallinity was recorded as 26.8%, 27.7%, and 29.0%, respectively, for alkali, wet-milling, and enzymatic samples. The low relative crystallinity of alkali and wet-milling methods suggests that the penetration of solvents and ions during extraction of starch possibly changed the original structure of amylose and amylopectin [32], and amylopectin is considered to be responsible for the formation of crystallites, which were possibly slightly and largely degraded at granule surface [29,30] in wet-milling and alkali methods of starch extraction.

#### 3.2.3. Lamellar Structure

The double logarithmic SAXS patterns, including scattering vector (q), lamellar distance (D), fractal dimension (α), and scattering intensity (I) of quinoa starch extracted by different methods (alkali, wet-milling, and enzymatic) are presented in Figure 2B and Table 2. The scattering patterns of quinoa starch extracted by alkali, wet-milling, and enzymatic methods exhibited a scattering vector peak at about 0.60 nm^−1^, which is assumed to represent the crystalline and amorphous regions and corresponds to lamellae repeat distance or Bragg spacing [33]. No differences were found in the values of scattering vector and lamellar distance among the three methods, whereas, the fractal dimension and scattering intensity of starch granules extracted by alkali (2.3 and 13.4 nm) and wet-milling (2.5 and 13.6 nm) were reduced compared to the enzymatic (2.6 and 17.8 nm) method. The higher scattering intensity of enzymatic extraction could be related to the higher electron density between amorphous and crystalline lamellae [23]. Moreover, the higher values of fractal dimension and scattering intensity obtained by the enzymatic method suggested that possibly the amylose content of quinoa starch was partially disrupted by alkali and wet-milling methods, which caused low scattering intensity [34]. The scattering intensity of enzymatically extracted starch possessed thicker and stronger semi-crystalline lamellae, which indicated that this method has a least effect on the semi-crystalline lamellae of starch.

#### 3.2.4. Ordered Short-Range Structure

The ATR-FTIR spectrum in other term is expressed as a short-range ordered structure or double-helical order observed in 2 µm depth of starch granules [35], and differs from the long-range ordered structure associated with packing of double helices often analyzed by XRD. The ATR-FTIR spectra of quinoa starch extracted by alkali, enzymatic, and wet-milling methods are presented in Figure 2C and Table 2. The absorbance bands at 1047, 1022, and 998 cm^−1^ were observed to be sensitive to starch conformation, which is related to amorphous regions of starch [36]. The absorbance ratio of 1047/1022 cm^−1^ and the molecular order of double helices are positively correlated with relative crystallinity than the absorbance ratio of 1047/998 cm^−1^ [37]. The enzymatic (0.9 and 1.2) and wet-milling (0.9 and 1.2) methods produced the higher ratios of 1047/1022 cm^−^^1^ and lower ratios of 1022/998 cm^−^^1^ than alkali (0.8 and 1.3). These results indicate that the alkali isolating method reduced the short-range molecular order of starch, which is consistent with the XRD data (Figure 2A). It is likely that the starch double helical structure of alkali was partially destroyed near the granule surface compared to the enzymatic and wet-milling methods, whose external granule regions were more ordered [36].

#### 3.2.5. Solid-State NMR

NMR is a very helpful technique to determine the structural differences of samples similar in nature. The helical structure (single and double helix) changes of quinoa starch extracted by alkali, enzymatic, and wet-milling methods were analyzed by solid-state NMR spectra, and the results are shown in Figure 2D and Table 2. Generally, two types of helices are observed in the starch granules of amylopectin side chains: packed helices, which are determined by both XRD and solid-state NMR spectroscopy, whereas unpacked helices are only detected by solid-state NMR spectra [38]. The ordered proportion of solid-state NMR spectroscopy is always higher than XRD. Peaks at 97–104, 78–84, and 58–63 ppm in the spectra are attributed to C1, C4, and C6 resonances, respectively, whereas, overlapping peaks at 67–77 ppm represent C2, C3, and C5 resonances [36]. The C1 resonance in spectra corresponds to the helical and crystalline structure of A-type starch. The amorphous and helical conformation changes of starch are placed in Table 2. The single (8.2%) and double (23.1%) helical proportions of the enzymatic method were observed higher along with lower amorphous proportion (68.7%) when compared with alkali (6.7%, 21.1%, and 72.2%) and wet-milling (7.3%, 21.2%, and 71.5%). The lower proportions of single and double helices of the wet-milling method could be due to the disruption of starch intramolecular hydrogen bonds, which increased the proportion of amorphous components, possibly caused by the milling process [39]. Similarly, starch granules extracted by the alkali method contained sodium hydroxide (0.3%) that likely contributed to solubilize protein matrix [26] and degrade starch [30]. Our results are in agreement with a previous study, in which authors compared the structure of chestnut fruit starches isolated by alkali and enzymatic methods, and found that the amorphous resonances of the alkali method were higher than the enzymatic, suggesting that amorphous compounds provide broad resonances because the distribution of local molecular environment gives rise to a broad distribution of chemical shifts [7].

#### 3.2.6. LCM-Raman Spectra

**Figure 3:**
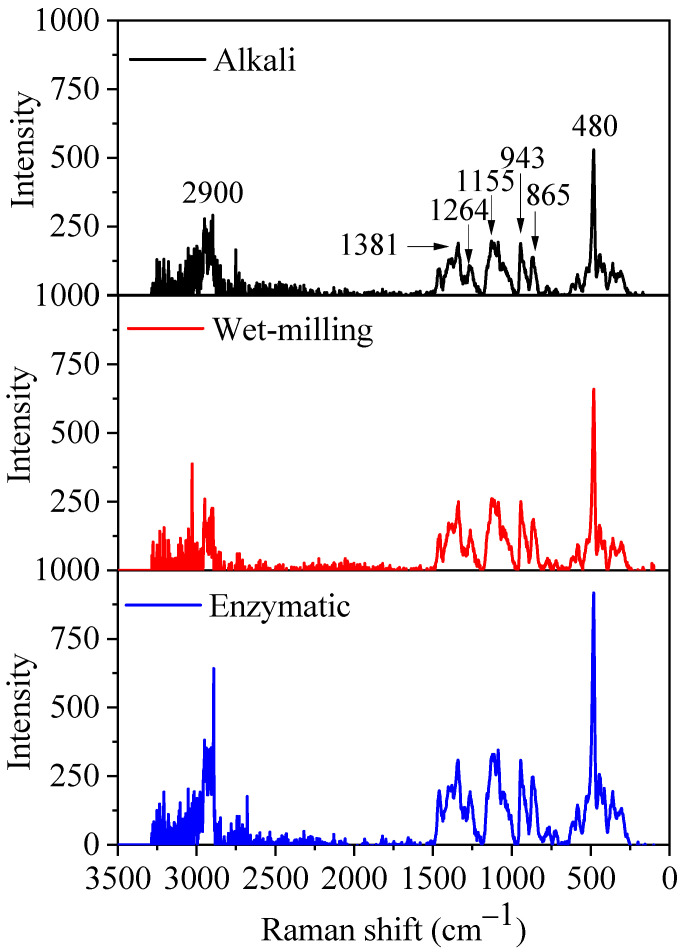
Raman spectrum of quinoa starch subjected to three different methods (alkali, wet-milling, and enzymatic).

The LCM-Raman spectra of quinoa starch extracted by alkali, wet-milling, and enzymatic methods are shown in Figure 3. Starch Raman spectra are generally classified into three main regions: <800 cm^−^^1^, 800–1500 cm^−^^1^ (fingerprint region), and 2800–3000 cm^−^^1^ (C–H stretch region). On the Raman spectra scale (0–3500 cm^−^^1^), the sharpening bands at 480, 865, 943, 1264, and 2900 cm^−^^1^ represent δ(CH2), ν_s_(C1–O–C4′), ν_s_(C1–O–C5), skeletal (C–C–O), and ν(C–H) modes, respectively, while the splitting bands at 1381 cm^−1^ and 1155 cm^−1^ are assigned to δ(C–OH) and δ(C–H) modes, and α-(C1–O–C4′) asymmetric stretch, respectively [40]. The narrow bands at 480 cm^−^^1^ and 2900 cm^−^^1^ represent the molecular order and its changes obtained from native quinoa starch granules with narrower bond energy distribution. The intensity of 480 cm^−^^1^ and 2900 cm^−^^1^ band was reduced in the starch samples extracted by alkali and wet-milling compared to the enzymatic method. Furthermore, the short-range ordered structural variations were studied with full-width at half-maximum (FWHM) at 480 cm^−^^1^ and its results are presented in Table 2. The FWHM value of the band at 480 cm^−^^1^ was recorded as 2.1, 1.7, and 1.5 for granules isolated by alkali, wet-milling, and enzymatic methods, the higher FWHM value of the alkali method confirming the disruption of the starch short-range ordered structure, consistent with XRD and FTIR results. Generally, the low FWHM values of 480 cm^−^^1^ at short-range scale have been suggested to have a more ordered molecular structure with high relative crystallinity [20,41].

#### 3.2.7. Molecular Weight and Molar Mass Ratio

The average molecular weight (*M*_w_), average radius of gyration (R_z_), and molar mass ratio (*M*_w_/*M*_n_) of quinoa starch extracted by different methods (alkali, wet-milling, and enzymatic) are presented in Table 2. The GPC-MALS analysis of enzymatically extracted granules revealed the higher *M*_w_ (1.58 × 10^7^ g/moL) and R_z_ (106.8 nm) than wet-milling (1.18 × 10^7^ g/moL and 101.1 nm) and alkali (1.13 × 10^5^ g/moL and 41.6 nm), whereas the *M*_w_/*M*_n_ of the enzymatic method (1.3) was observed to be lower compared to alkali (2.7), but slightly higher than wet-milling (1.2). The higher *M*_w_ and R_z_ of the enzymatic method indicate the greater molecules with a larger molecular size of the amylopectin, whereas the low *M*_w_ and R_z_ values of the alkali and wet-milling methods suggest that amylose and amylopectin molecules of starch granules were possibly disintegrated into less super molecular components due to the high pH value in the alkali method [42], as well as high milling intensity [43], which eventually damage the morphological, crystalline, and molecular structure of starch. Furthermore, Zhao et al. soaked rice powder in 0.45% sodium metabisulfite for 18 h at 4 °C, followed by mixing with protease for overnight to extract rice starch and observed the largest amylopectin molecular weight, which suggested that protease had the least effect on extracted starch [15].

### 3.3. Functional Properties

#### 3.3.1. Thermal Properties

The results of quinoa starch thermal properties extracted by alkali, wet-milling, and enzymatic methods are shown in Table 3. The DSC onset temperature (*T*_o_), peak temperature (*T*_p_), and conclusion temperature (*T*_c_) of gelatinization represent the hydrothermal stability of the starch crystalline structure, and enthalpy change (Δ*H*) corresponds to the loss of the starch granule molecular (double-helical) structure [44]. The *T*_o_, *T*_p_, *T*_c_, and Δ*H* of the enzymatic (82.1, 83.8, 86.3 °C, and 6.8 J/g) extraction method were significantly higher than wet-milling (66.3, 70.0, 76.2 °C, and 6.7 J/g), and alkali (61.7, 67.8, 75.9 °C, and 5.4 J/g). All the above characteristic temperatures and Δ*H* exhibited the order of enzymatic > wet-milling > alkali. The results contrast the previous study of Correia et al., who studied the thermal properties of Portuguese nuts starches isolated by alkaline and enzymatic methods and observed similar gelatinization temperatures (61.5–63.0 °C) [45]. However, the differences observed in our study could be due to the granule size, microstructure, order of crystalline regions, ratio of amylose to amylopectin, and minor components [45]. The higher *T*_o_, *T*_p_, *T*_c_, and Δ*H* exhibited by the enzymatic method containing large starch granules inhibited the gelatinization, water absorption, and eventually lowered granule swelling, which were most likely exerted by the minor components (i.e., protein and lipids) [46,47]. The *T*_o_, *T*_p_, *T*_c_, and Δ*H* of the enzymatic method are positively correlated to their particle size D_50_ (Table 1). A similar study conducted on large and small wheat starch granules suggested that large granules showed higher *T*_o_, *T*_p_, *T*_c_, and Δ*H* with high relative crystallinity than compared to small granules [48]. Another study conducted on rice starch granules showed that small particles were observed with low *T*_o_, *T*_p_, *T*_c_, and Δ*H* [49]. The gelatinization temperatures of alkali and wet-milling methods highly ranged from onset to end set temperatures, suggesting the presence of crystallites of varying stability in the granule crystalline domains, whereas enzymatically extracted starch of the higher *T*_o_, *T*_p_, and Δ*H* with narrow temperature range suggests that it possibly has higher molecular order [45]. Thus, enzymatic method better preserves the molecular order.

#### 3.3.2. Textural Properties

Textural properties are described in terms such as hard, soft, liquid, solid, rough, smooth, creamy, crispy, lumpy, gritty, etc., to assess the quality of a product, which is related to density, viscosity, surface tension, and other physical properties of the specific product [50]. Table 3 shows the textural properties (hardness, springiness, gumminess, chewiness, and resilience) of quinoa starch extracted by alkali, wet-milling, and enzymatic methods. The enzymatic method of extraction showed the highest values of hardness (238.8 N), springiness (0.8), gumminess (105.6 N), chewiness (80.2 N), and resilience (0.2) compared with the wet-milling (192.7 N, 0.6, 91.3 N, 54.9 N, and 0.1) and alkali (160.5 N, 0.3, 41.5 N, 13.5 N, and 0.1) methods. On the other hand, the alkali had poor textural properties. The highest textural values of the enzymatic method suggest the presence of less compact gas cells with high specific volume [51] and elasticity [22]. We assume that the starch granules extracted by enzymatic method probably had rigid structure with higher proportion of long branch chains of amylopectin [52]. Furthermore, a study conducted on yam starch and observed the higher texture parameters with larger starch granule size and lower amylose content, whereas lower texture properties in counterparts was suggested due to partial hydrolysis of granules [53].

#### 3.3.3. *In Vitro* Digestion Properties

The impact of starch extraction methods (alkali, wet-milling, and enzymatic) on *in vitro* digestion properties (RDS, SDS, and RS) of raw (uncooked) and cooked quinoa starch was investigated, and the obtained results are presented in Table 3. During the starch hydrolysis of 0–120 min, uncooked starch of the enzymatic extraction method showed the lowest RDS (77.1%) and SDS (7.5%), and the highest RS (15.4%) contents compared to alkali (86.6%, 5.2%, and 8.2%) and wet-milling (85.4%, 5.9%, and 8.6%). Similarly, the hydrolysis of cooked starch extracted by wet-milling and alkali was observed with the highest RDS (87.7% and 88.3%) and SDS (4.1% and 3.9%), and the lowest RS (7.6% and 7.9%) compared to the enzymatic method (81.9%, 4.8%, and 13.9%). The RDS, SDS, and RS contents of cooked starch were observed significantly higher than those of uncooked starch. The results suggested that the enzymatic method of starch extraction resisted more *in vitro* digestion in both uncooked and cooked conditions. Previous studies have reported that digestion of starch is affected by size, crystallinity, polymerization, non-starch components, and amylose/amylopectin ratio [54,55]. The resistance of the enzymatic extraction method might be due to its large granule size, which limited gelatinization by inhibiting hydration and swelling, prevented the enzyme from substrate accessibility, and eventually reduced starch digestion [56]. The small amounts of protein and lipids surrounded by starch possibly played their roles in reducing starch swelling [57]. Furthermore, SDS and RS contents are positively correlated with enthalpy and relative crystallinity, and higher crystallinity and enthalpy reduce starch digestion [58].

#### 3.3.4. Principal Component Analysis (PCA)

**Figure 4:**
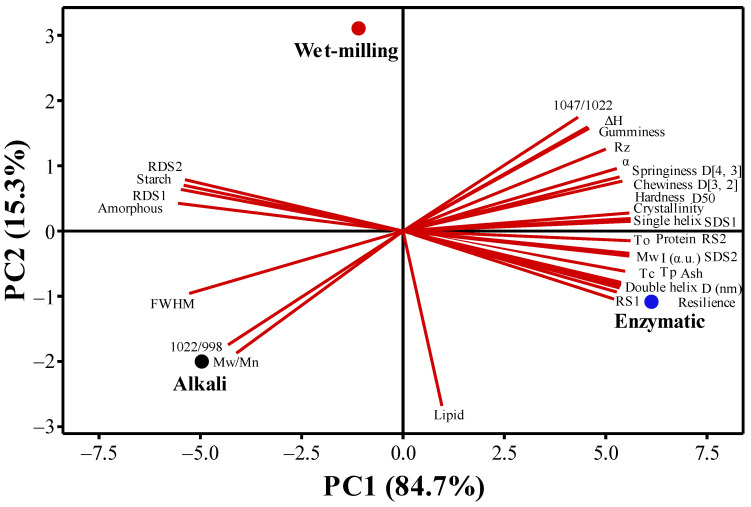
The PC1 and PC2 double dimensional plots of quinoa starch extracted by alkali, wet-milling, and enzymatic methods.

The multi-scale structure and functional characteristics of quinoa starch obtained by different methods of extraction (alkali, wet-milling, and enzymatic) were compared by PCA. The similarities, differences, and distribution of quality attributes defined by the first and second PCA dimensions of the starch samples isolated by alkali, wet-milling, and enzymatic methods are shown in Figure 4. The PC1 principal component showed the majority of variability with 84.7% and PC2 exhibited 15.3% variability, while the sum of PC1 and PC2 was observed to have 100% of the variations among starch samples. The starch samples extracted by alkali and wet-milling methods were observed on the negative side of the PC1, while the enzymatic method remained on the positive side of the PC1. According to the principal component analysis score (Table 4), the quinoa starch extracted by the enzymatic method has better multi-scale structure and functional characteristics.

## 4. Conclusions

In this study, quinoa starch was extracted by alkali, wet-milling, and enzymatic methods to investigate the impact of extraction methods on starch multi-scale structures and functional properties. The morphological, compositional, multi-scale structural (i.e., XRD, SAXS, FTIR, NMR, LCM-Raman and GPC), thermal, textural, and *in vitro* digestion analyses were subsequently performed. Starch granules extracted by the enzymatic method exhibited the larger size of aggregated granules, higher protein content, relative crystallinity, semi-crystalline lamellae, absorbance ratios of 1047/1022 and 1022/998 cm^−1^, helical structure content, M_w_, and lower FWHM ratio at 480 cm^−1^ compared with wet-milling and alkali methods. Furthermore, the enzymatic method increased the texture properties (i.e., hardness, springiness, gumminess, chewiness, and resilience), SDS, and RS content. The corresponding methods, on the other hand, reduced the gelatinization and RDS. From these results, it is concluded that extraction methods induce significant structural differences in starch granules. Enzymatic extraction is the more appropriate method to retain the intact structure of starch, whereas the alkali method is highly effective in the extraction and purification of starch, which may lead to different functional properties and industrial applications.

## Figures and Tables

**Table 1:** Proximate composition and particle size of quinoa starch isolated by three different methods (alkali, wet-milling, and enzymatic) ^A^.

Variables	Alkali	Wet-milling	Enzymatic
Composition (DB) ^B^			
Total starch content (%)	98.7 ± 1.5 ^a^	98.6 ± 1.1 ^a^	97.1 ± 0.8 ^a^
Protein content (%)	0.6 ± 0.1 ^c^	0.9 ± 0.1 ^b^	2.4 ± 0.0 ^a^
Lipid content (%)	0.5 ± 0.0 ^a^	0.3 ± 0.0 ^b^	0.5 ± 0.0 ^a^
Ash content (%)	0.1 ± 0.0 ^b^	0.1 ± 0.0 ^b^	0.2 ± 0.0 ^a^
Granule size distribution ^C^			
D_50_ (μm)	27.6 ± 4.5 ^c^	34.9 ± 0.4 ^b^	44.1 ± 3.2 ^a^
D_[4, 3]_ (μm)	34.5 ± 3.6 ^b^	39.9 ± 2.8 ^b^	47.7 ± 0.3 ^a^
D_[3, 2]_ (μm)	6.2 ± 0.7 ^c^	7.4 ± 0.2 ^b^	11.3 ± 0.7 ^a^

^A^ The data are means and standard deviation of double experiments. The values in the row with different superscripted letters are significant (*p* < 0.05). ^B^ DB, dry basis. ^C^ D_50,_ mean diameter; D_[4, 3]_, volume-weighted mean diameter; and D_[3, 2]_, surface-weighted mean diameter of quinoa starch granules.

**Table 2:** Multi-scale structure and molecular weight changes in quinoa starch isolated by three different methods (alkali, wet-milling, and enzymatic) ^A^.

Structural Characteristics ^B^	Variables ^C^	Alkali	Wet-milling	Enzymatic
XRD	Relative crystallinity (%)	26.8 ± 0.2 ^b^	27.7 ± 0.5 ^b^	29.0 ± 0.3 ^a^
SAXS	α	2.3 ± 0.3 ^b^	2.5 ± 0.2 ^a^	2.6 ± 0.1 ^a^
	I (α.u.)	13.4 ± 0.7 ^b^	13.6 ± 0.3 ^b^	17.8 ± 0.5 ^a^
	q (1/nm)	0.6 ± 0.0 ^a^	0.6 ± 0.0 ^a^	0.6 ± 0.0 ^a^
	D (nm)	10.1 ± 0.1 ^a^	10.0 ± 0.1 ^a^	10.1 ± 0.2 ^a^
ATR-FTIR	Ratio of 1047/1022 cm^−1^	0.8 ± 0.0 ^b^	0.9 ± 0.0 ^a^	0.9 ± 0.0 ^a^
	Ratio of 1022/998 cm^−1^	1.3 ± 0.0 ^a^	1.2 ± 0.0 ^b^	1.2 ± 0.0 ^b^
^13^C CP/MAS NMR	Single helix (%)	6.7 ± 0.2 ^b^	7.3 ± 0.3 ^b^	8.2 ± 0.0 ^a^
	Double helix (%)	21.1 ± 0.2 ^b^	21.2 ± 0.2 ^b^	23.1 ± 0.4 ^a^
	Amorphous (%)	72.2 ± 0.3 ^a^	71.5 ± 0.1 ^a^	68.7 ± 0.3 ^a^
LCM-Raman	FWHM at 480 cm^−1^	2.1 ± 0.0 ^a^	1.7 ± 0.0 ^b^	1.5 ± 0.0 ^c^
GPC-MALS	Average *M*_w_ (g/moL)	1.13 × 10^5^ (2%)	1.18 × 10^7^ (3%)	1.58 × 10^7^ (3%)
	Average R_z_ (nm)	41.6 (4%)	101.1 (1%)	106.8 (1%)
	*M*_w_/*M*_n_	2.7 (10%)	1.2 (6%)	1.3 (5%)

^A^ Values are means of two replicates with standard deviation, means in row with same superscripted letters are not significant (*p* > 0.05). ^B^ Multi-scale structure analyses were performed by XRD, X-ray diffraction; ATR-FTIR, attenuated total reflectance-Fourier transform infrared spectroscopy; LCM-Raman, laser confocal microscopy-Raman; ^13^C CP/MAS NMR, carbon-13 cross-polarization/magic angle spinning nuclear magnetic resonance; and GPC-MALS, Gel permeation chromatography coupled with multi-angle light scattering. ^C^ Multi-scale variables including α, fractal dimension; I, scattering intensity; q, scattering vector; D, lamellar distance; FWHM, full-width at half-maximum; *M*_w_, molecular weight; R_z_, radius of gyration; and *M*_w_/*M*_n,_ mass molar ratio are shown.

**Table 3:** Thermal, textural, and *in vitro* digestion properties of quinoa starch isolated by three different methods (alkali, wet-milling, and enzymatic) ^A^.

Variables	Alkali	Wet-milling	Enzymatic
Thermal properties ^B^			
*T*_o_ (°C)	61.7 ± 0.9 ^c^	66.3 ± 0.9 ^b^	82.1 ± 1.2 ^a^
*T*_p_ (°C)	67.8 ± 0.5 ^b^	70.0 ± 0.5 ^b^	83.8 ± 0.5 ^a^
*T*_c_ (°C)	75.9 ± 0.5 ^b^	76.2 ± 0.9 ^b^	86.3 ± 0.2 ^a^
Δ*H* (J/g)	5.4 ± 0.8 ^b^	6.7 ± 0.5 ^a^	6.8 ± 0.5 ^a^
Textural properties			
Hardness (N)	160.5 ± 2.1 ^c^	192.7 ± 2.3 ^b^	238.8 ± 2.4 ^a^
Springiness	0.3 ± 0.0 ^c^	0.6 ± 0.0 ^b^	0.8 ± 0.0 ^a^
Gumminess (N)	41.5 ± 0.6 ^c^	91.3 ± 0.8 ^b^	105.6 ± 1.1 ^a^
Chewiness (N)	13.5 ± 0.3 ^c^	54.9 ± 0.5 ^b^	80.2 ± 0.9 ^a^
Resilience	0.1 ± 0.0 ^b^	0.1 ± 0.0 ^b^	0.2 ± 0.0 ^a^
*In vitro* digestion of raw (uncooked) samples ^C^			
RDS (%)	86.6 ± 0.6 ^a^	85.4 ± 0.5 ^a^	77.1 ± 0.4 ^b^
SDS (%)	5.2 ± 0.6 ^b^	5.9 ± 0.4 ^b^	7.5 ± 0.8 ^a^
RS (%)	8.2 ± 0.0 ^b^	8.6 ± 0.9 ^b^	15.4 ± 0.4 ^a^
*In vitro* digestion of cooked samples ^C^			
RDS (%)	88.3 ± 0.5 ^a^	87.7 ± 0.2 ^a^	81.9 ± 2.0 ^b^
SDS (%)	3.9 ± 0.4 ^b^	4.1 ± 0.1 ^a^	4.8 ± 0.0 ^b^
RS (%)	7.9 ± 0.9 ^b^	7.6 ± 0.2 ^b^	13.9 ± 1.8 ^a^

^A^ The data are means and standard deviation of double experiments. Means in the row with different superscripted letters are significant (*p* < 0.05). ^B^ The terms *T*_o_, *T*_p_, *T*_c_, and Δ*H* are represent the onset, peak, conclusion temperatures, and enthalpy change. ^C^ RDS, SDS, and RS are denoted as rapidly digestible starch, slowly digestible starch, and resistant starch.

**Table 4:** Principal component analysis score of quinoa starch extracted by alkali, wet-milling, and enzymatic method.

Variables	Alkali	Wet-Milling	Enzymatic
y_1_	−4.98343	−1.10765	6.09108
y_2_	−2.01439	3.09893	1.05454
y	6.99782	1.99128	8.9891

## Data Availability

Data is contained within the article.

## Abbreviations

SPSS: Statistical product and service solution; GOPOD, glucose oxidase-peroxidase reagent; DSC, differential scanning calorimetry; ANOVA, analysis of variance; SEM, scanning electron microscopy; XRD, X-ray diffraction; ATR-FTIR, attenuated total reflectance-fourier transformed infrared; SAXS, small-angle X-ray scattering; LCM-Raman, laser confocal micro-Raman; MAS NMR, magic-angle spinning nuclear magnetic resonance; GPC-MALS, gel permeation chromatography coupled with multi-angle light scattering; FWHM, full-width at half-maximum; RDS, rapidly digestible starch; SDS, slowly digestible starch; RS, resistant starch; PCA, principal component analysis.
